# 1-(Biphenyl-4-ylcarbon­yl)-3-(2-chloro-4-nitro­phen­yl)thio­urea

**DOI:** 10.1107/S1600536812016686

**Published:** 2012-04-21

**Authors:** M. Sukeri M. Yusof, Bohari M. Yamin, Nurziana Ngah

**Affiliations:** a Department of Chemical Sciences, Faculty of Science and Technology, Universiti Malaysia Terengganu, Menggabang Telipot, 21030 Kuala Terengganu, Malaysia; b School of Chemical Sciences and Food Technology, Universiti Kebangsaan Malaysia, UKM 43600 Bangi Selangor, Malaysia; c Kulliyyah of Science, International Islamic University Malaysia, Bandar Indera Mahkota, 25200 Kuantan, Pahang, Malaysia

## Abstract

The benzene rings of the biphenyl group in the title compound, C_20_H_14_ClN_3_O_3_S, are nearly coplanar [maximum deviation = 0.20 (3) Å]. The mean plane of the biphenyl group forms a dihedral angle of 5.24 (7)° with the aromatic ring of the nitro­chloro­benzene group. Intra­molecular N—H⋯Cl, N—H⋯O and C—H⋯S hydrogen bonds stabilize the *cis–trans* conformation of the mol­ecule. In the crystal, mol­ecules are linked by C—H⋯O and C—H⋯S hydrogen bonds into mutually inter­woven corrugated layers parallel to (10-2).

## Related literature

For a related structure, see: Yusof *et al.* (2011 ▶). For bond-length data, see: Allen *et al.* (1987 ▶).
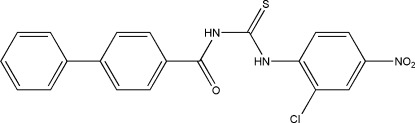


## Experimental

### 

#### Crystal data

C_20_H_14_ClN_3_O_3_S
*M* *_r_* = 411.85
Monoclinic, 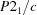

*a* = 10.889 (2) Å
*b* = 5.4502 (10) Å
*c* = 30.532 (5) Å
β = 99.202 (4)°
*V* = 1788.6 (6) Å^3^
*Z* = 4
Mo *K*α radiation
μ = 0.36 mm^−1^
*T* = 298 K
0.35 × 0.13 × 0.08 mm

#### Data collection

Bruker SMART APEX CCD area-detector diffractometer
Absorption correction: multi-scan (*SADABS*; Bruker, 2000 ▶) *T*_min_ = 0.885, *T*_max_ = 0.972
9537 measured reflections
3154 independent reflections
2704 reflections with *I* > 2/s(*I*)
*R*_int_ = 0.026

#### Refinement

*R*[*F* ^2^ > 2σ(*F* ^2^)] = 0.049
*wR*(*F* ^2^) = 0.137
*S* = 1.16
3154 reflections
253 parameters
3 restraints
H-atom parameters constrained
Δρ_max_ = 0.25 e Å^−3^
Δρ_min_ = −0.24 e Å^−3^

### 

Data collection: *SMART* (Bruker, 2000 ▶); cell refinement: *SAINT* (Bruker, 2000 ▶); data reduction: *SAINT*; program(s) used to solve structure: *SHELXS97* (Sheldrick, 2008 ▶); program(s) used to refine structure: *SHELXL97* (Sheldrick, 2008 ▶); molecular graphics: *SHELXTL* (Sheldrick, 2008 ▶); software used to prepare material for publication: *SHELXTL*, *PARST* (Nardelli, 1995 ▶) and *PLATON* (Spek, 2009 ▶).

## Supplementary Material

Crystal structure: contains datablock(s) global, I. DOI: 10.1107/S1600536812016686/rz2737sup1.cif

Structure factors: contains datablock(s) I. DOI: 10.1107/S1600536812016686/rz2737Isup2.hkl

Supplementary material file. DOI: 10.1107/S1600536812016686/rz2737Isup3.cml

Additional supplementary materials:  crystallographic information; 3D view; checkCIF report

## Figures and Tables

**Table 1:** Hydrogen-bond geometry (Å, °)

*D*—H⋯*A*	*D*—H	H⋯*A*	*D*⋯*A*	*D*—H⋯*A*
N2—H2*A*⋯Cl1	0.86	2.43	2.938 (2)	118
N2—H2*A*⋯O1	0.86	1.87	2.608 (3)	143
C16—H16*A*⋯S1	0.93	2.53	3.208 (3)	130
C10—H10*A*⋯O3^i^	0.93	2.57	3.354 (4)	142
C17—H17*A*⋯S1^ii^	0.93	2.77	3.673 (3)	165

Symmetry codes: (i) 
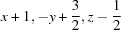
; (ii) 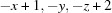.

**Fig. 1.**
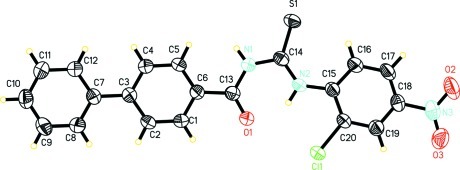
The molecular structure of the title compound with displacement ellipsods drawn at the 50% probability level.

**Fig. 2.**
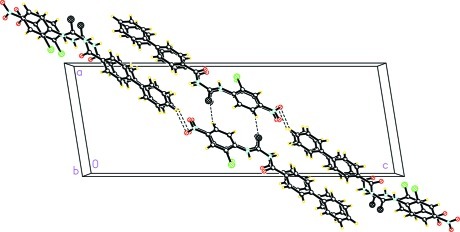
The crystal packing of the title compound viewed down the b axis. Intermolecular hydrogen bonds are shown as dashed lines.

## supplementary crystallographic information

### Comment

The title compound, (I), is similiar to the previously reported compound 1-(biphenyl-4-yl-carbonyl)-3-(4-nitrophenyl)thiourea (II) (Yusof *et al.*, 2011) except for the presence of a chlorine atom at 2-position of the nitrobenzene ring. The bond lengths and angles are in normal ranges (Allen *et al.* 1987) and comparable to those reported for (II). The molecule (Fig. 1) is essentially planar, with a maximum deviation of 0.076 (2) Å for atom N1. The introduction of a chlorine substituent at the 2-position of the nitrobenzene ring makes the two benzene rings in biphenyl group nearly coplanar, forming a dihedral angle of 1.02 (14)° compared to that of 40.11 (15)° observed in II. The thiourea moiety makes dihedral angle of 6.15 (10)° and 0.92 (10)° with the C1—C6 and C15—C20 rings, respectively, compared to the corresponding angles of 16.14 (13)° and 17.75 (14)° in II. The *cis-trans* conformation of the molecule is stabilized by intramolecular N2—H2A···Cl1, N2—H2A···O1 and C16—H16A···S1 hydrogen interactions (Table 1). In the crystal structure (Fig. 2), the molecules interact through intermolecular C—H···O and C—H···S hydrogen bonds to form mutually interwoven corrugated layers parallel to the (1 0 -2) plane.

### Experimental

An acetone (30 ml) solution of 2-chloro-4-nitroaniline (1.60 g, 9.5 mmol) was added to a round-bottom flask containing 4-biphenylcarbonyl chloride (2.00 g, 9.5 mmol) and ammonium thiocyanate (0.70 g, 9.5 mmol). The mixture was refluxed for 2.5 h then filtered off and left to evaporate at room temperature. The yellowish precipitate obtained was washed with water and cold ethanol. Yellowish crystals suitable for X-ray analysis were obtained by recrystallization of the precipitate in DMSO.

### Refinement

All H atoms were positioned geometrically and refined using a riding model with C—H = 0.93 Å and N—H = 0.86 Å, and with *U*_iso_(H) = 1.2 *U*_eq_(C, N). A rigid body restraint (DELU in *SHELXL-97*; Sheldrick, 2008) was applied for atoms N3, O2 and O3.

### Crystal data

**Table:**

C_20_H_14_ClN_3_O_3_S	*F*(000) = 848
*M**_r_* = 411.85	*D*_x_ = 1.529 Mg m^−^^3^
Monoclinic, *P*2_1_/*c*	Mo *K*α radiation, λ = 0.71073 Å
Hall symbol: -P 2ybc	Cell parameters from 1235 reflections
*a* = 10.889 (2) Å	θ = 1.9–25.0°
*b* = 5.4502 (10) Å	µ = 0.36 mm^−^^1^
*c* = 30.532 (5) Å	*T* = 298 K
β = 99.202 (4)°	Slab, yellow
*V* = 1788.6 (6) Å^3^	0.35 × 0.13 × 0.08 mm
*Z* = 4	

### Data collection

**Table:**

Bruker SMART APEX CCD area-detector diffractometer	3154 independent reflections
Radiation source: fine-focus sealed tube	2704 reflections with *I* > 2/s(*I*)
Graphite monochromator	*R*_int_ = 0.026
Detector resolution: 83.66 pixels mm^-1^	θ_max_ = 25.0°, θ_min_ = 1.9°
ω scan	*h* = −11→12
Absorption correction: multi-scan (*SADABS*; Bruker, 2000)	*k* = −6→6
*T*_min_ = 0.885, *T*_max_ = 0.972	*l* = −35→36
9537 measured reflections	

### Refinement

**Table:**

Refinement on *F*^2^	Primary atom site location: structure-invariant direct methods
Least-squares matrix: full	Secondary atom site location: difference Fourier map
*R*[*F*^2^ > 2σ(*F*^2^)] = 0.049	Hydrogen site location: inferred from neighbouring sites
*wR*(*F*^2^) = 0.137	H-atom parameters constrained
*S* = 1.16	*w* = 1/[σ^2^(*F*_o_^2^) + (0.0709*P*)^2^ + 0.5419*P*] where *P* = (*F*_o_^2^ + 2*F*_c_^2^)/3
3154 reflections	(Δ/σ)_max_ = 0.002
253 parameters	Δρ_max_ = 0.25 e Å^−^^3^
3 restraints	Δρ_min_ = −0.24 e Å^−^^3^

### Special details

**Table:**

Geometry. All e.s.d.'s (except the e.s.d. in the dihedral angle between two l.s. planes) are estimated using the full covariance matrix. The cell e.s.d.'s are taken into account individually in the estimation of e.s.d.'s in distances, angles and torsion angles; correlations between e.s.d.'s in cell parameters are only used when they are defined by crystal symmetry. An approximate (isotropic) treatment of cell e.s.d.'s is used for estimating e.s.d.'s involving l.s. planes.
Refinement. Refinement of *F*^2^ against ALL reflections. The weighted *R*-factor *wR* and goodness of fit *S* are based on *F*^2^, conventional *R*-factors *R* are based on *F*, with *F* set to zero for negative *F*^2^. The threshold expression of *F*^2^ > σ(*F*^2^) is used only for calculating *R*-factors(gt) *etc*. and is not relevant to the choice of reflections for refinement. *R*-factors based on *F*^2^ are statistically about twice as large as those based on *F*, and *R*- factors based on ALL data will be even larger.

### Fractional atomic coordinates and isotropic or equivalent isotropic displacement parameters (Å^2^)

**Table:**

	*x*	*y*	*z*	*U*_iso_*/*U*_eq_	
S1	0.66457 (7)	0.06984 (14)	0.93137 (2)	0.0553 (3)	
Cl1	0.88661 (6)	0.87141 (13)	1.03030 (2)	0.0511 (2)	
O1	0.9472 (2)	0.6794 (4)	0.93384 (7)	0.0691 (7)	
O2	0.5004 (3)	0.4603 (5)	1.13921 (9)	0.0849 (8)	
O3	0.6132 (2)	0.7850 (5)	1.15220 (7)	0.0701 (6)	
N1	0.8315 (2)	0.3541 (4)	0.90589 (7)	0.0449 (5)	
H1A	0.8162	0.2613	0.8829	0.054*	
N2	0.7806 (2)	0.4647 (4)	0.97322 (7)	0.0456 (5)	
H2A	0.8366	0.5731	0.9709	0.055*	
N3	0.5772 (2)	0.6060 (5)	1.12973 (9)	0.0559 (6)	
C1	1.0767 (3)	0.7043 (5)	0.86344 (10)	0.0538 (7)	
H1B	1.0856	0.8296	0.8844	0.065*	
C2	1.1482 (3)	0.7078 (5)	0.83008 (9)	0.0509 (7)	
H2B	1.2040	0.8359	0.8290	0.061*	
C3	1.1390 (2)	0.5250 (5)	0.79806 (8)	0.0374 (6)	
C4	1.0527 (3)	0.3419 (5)	0.80119 (9)	0.0520 (7)	
H4A	1.0432	0.2171	0.7801	0.062*	
C5	0.9802 (3)	0.3377 (5)	0.83435 (9)	0.0509 (7)	
H5A	0.9228	0.2119	0.8351	0.061*	
C6	0.9919 (2)	0.5184 (5)	0.86650 (8)	0.0394 (6)	
C7	1.2173 (2)	0.5270 (5)	0.76232 (8)	0.0393 (6)	
C8	1.3045 (3)	0.7076 (6)	0.75971 (10)	0.0609 (8)	
H8A	1.3153	0.8315	0.7809	0.073*	
C9	1.3767 (3)	0.7083 (7)	0.72601 (11)	0.0681 (9)	
H9A	1.4342	0.8333	0.7248	0.082*	
C10	1.3638 (3)	0.5275 (6)	0.69487 (9)	0.0551 (8)	
H10A	1.4127	0.5274	0.6725	0.066*	
C11	1.2787 (3)	0.3470 (7)	0.69684 (11)	0.0661 (9)	
H11A	1.2691	0.2228	0.6757	0.079*	
C12	1.2060 (3)	0.3469 (6)	0.73013 (10)	0.0570 (8)	
H12A	1.1481	0.2222	0.7308	0.068*	
C13	0.9227 (2)	0.5278 (5)	0.90462 (9)	0.0439 (6)	
C14	0.7603 (2)	0.3068 (5)	0.93919 (8)	0.0404 (6)	
C15	0.7273 (2)	0.4852 (5)	1.01169 (8)	0.0408 (6)	
C16	0.6357 (3)	0.3307 (6)	1.02337 (10)	0.0543 (7)	
H16A	0.6067	0.2002	1.0050	0.065*	
C17	0.5876 (3)	0.3692 (6)	1.06191 (10)	0.0547 (7)	
H17A	0.5274	0.2636	1.0696	0.066*	
C18	0.6284 (2)	0.5623 (5)	1.08869 (9)	0.0446 (6)	
C19	0.7191 (2)	0.7181 (5)	1.07878 (8)	0.0449 (6)	
H19A	0.7467	0.8485	1.0974	0.054*	
C20	0.7686 (2)	0.6773 (5)	1.04071 (8)	0.0396 (6)	

### Atomic displacement parameters (Å^2^)

**Table:**

	*U*^11^	*U*^22^	*U*^33^	*U*^12^	*U*^13^	*U*^23^
S1	0.0593 (5)	0.0552 (5)	0.0514 (4)	−0.0271 (4)	0.0085 (3)	−0.0054 (3)
Cl1	0.0524 (4)	0.0538 (4)	0.0498 (4)	−0.0219 (3)	0.0157 (3)	−0.0058 (3)
O1	0.0819 (15)	0.0638 (14)	0.0722 (14)	−0.0380 (12)	0.0450 (12)	−0.0317 (12)
O2	0.0952 (18)	0.0893 (18)	0.0836 (16)	−0.0164 (13)	0.0550 (15)	0.0070 (14)
O3	0.0645 (14)	0.0899 (16)	0.0605 (13)	0.0008 (11)	0.0238 (11)	−0.0129 (12)
N1	0.0453 (12)	0.0477 (13)	0.0434 (12)	−0.0120 (10)	0.0128 (10)	−0.0082 (10)
N2	0.0458 (13)	0.0420 (12)	0.0526 (13)	−0.0151 (10)	0.0190 (10)	−0.0077 (11)
N3	0.0498 (14)	0.0645 (16)	0.0577 (14)	0.0066 (11)	0.0216 (11)	0.0093 (11)
C1	0.0617 (18)	0.0461 (17)	0.0583 (17)	−0.0176 (14)	0.0241 (14)	−0.0175 (14)
C2	0.0548 (17)	0.0448 (16)	0.0573 (17)	−0.0188 (13)	0.0219 (13)	−0.0077 (13)
C3	0.0366 (13)	0.0363 (13)	0.0390 (13)	0.0025 (10)	0.0051 (10)	0.0045 (11)
C4	0.0615 (18)	0.0478 (17)	0.0504 (16)	−0.0162 (14)	0.0201 (13)	−0.0125 (13)
C5	0.0573 (17)	0.0461 (16)	0.0534 (16)	−0.0211 (13)	0.0216 (13)	−0.0096 (13)
C6	0.0393 (13)	0.0382 (14)	0.0410 (13)	−0.0018 (11)	0.0076 (11)	0.0008 (11)
C7	0.0386 (13)	0.0429 (14)	0.0361 (12)	0.0016 (11)	0.0049 (10)	0.0066 (11)
C8	0.071 (2)	0.061 (2)	0.0552 (17)	−0.0232 (16)	0.0239 (15)	−0.0135 (15)
C9	0.073 (2)	0.075 (2)	0.0625 (19)	−0.0270 (18)	0.0315 (16)	−0.0033 (17)
C10	0.0517 (17)	0.072 (2)	0.0442 (15)	0.0002 (15)	0.0169 (13)	0.0032 (15)
C11	0.070 (2)	0.074 (2)	0.0601 (18)	−0.0131 (18)	0.0278 (16)	−0.0217 (17)
C12	0.0601 (18)	0.0551 (18)	0.0606 (17)	−0.0152 (14)	0.0240 (14)	−0.0149 (15)
C13	0.0427 (14)	0.0406 (15)	0.0500 (15)	−0.0056 (12)	0.0118 (12)	−0.0052 (13)
C14	0.0337 (13)	0.0414 (15)	0.0452 (14)	−0.0030 (11)	0.0036 (10)	0.0034 (12)
C15	0.0375 (14)	0.0404 (14)	0.0461 (14)	−0.0030 (11)	0.0113 (11)	0.0016 (12)
C16	0.0545 (17)	0.0507 (17)	0.0624 (18)	−0.0163 (14)	0.0236 (14)	−0.0086 (14)
C17	0.0513 (16)	0.0541 (18)	0.0645 (18)	−0.0134 (14)	0.0265 (14)	0.0032 (15)
C18	0.0389 (14)	0.0507 (16)	0.0463 (14)	0.0028 (12)	0.0133 (11)	0.0062 (13)
C19	0.0425 (14)	0.0482 (16)	0.0441 (14)	−0.0021 (12)	0.0078 (11)	0.0005 (12)
C20	0.0343 (13)	0.0416 (15)	0.0439 (13)	−0.0056 (11)	0.0097 (10)	0.0027 (11)

### Geometric parameters (Å, º)

**Table:**

S1—C14	1.653 (3)	C5—H5A	0.9300
Cl1—C20	1.733 (3)	C6—C13	1.485 (4)
O1—C13	1.214 (3)	C7—C8	1.379 (4)
O2—N3	1.221 (3)	C7—C12	1.380 (4)
O3—N3	1.221 (3)	C8—C9	1.391 (4)
N1—C13	1.377 (3)	C8—H8A	0.9300
N1—C14	1.397 (3)	C9—C10	1.361 (5)
N1—H1A	0.8600	C9—H9A	0.9300
N2—C14	1.340 (3)	C10—C11	1.360 (5)
N2—C15	1.395 (3)	C10—H10A	0.9300
N2—H2A	0.8600	C11—C12	1.384 (4)
N3—C18	1.470 (4)	C11—H11A	0.9300
C1—C2	1.377 (4)	C12—H12A	0.9300
C1—C6	1.384 (4)	C15—C16	1.395 (4)
C1—H1B	0.9300	C15—C20	1.398 (4)
C2—C3	1.388 (4)	C16—C17	1.378 (4)
C2—H2B	0.9300	C16—H16A	0.9300
C3—C4	1.385 (4)	C17—C18	1.363 (4)
C3—C7	1.488 (3)	C17—H17A	0.9300
C4—C5	1.380 (4)	C18—C19	1.373 (4)
C4—H4A	0.9300	C19—C20	1.375 (3)
C5—C6	1.382 (4)	C19—H19A	0.9300
			
C13—N1—C14	129.3 (2)	C8—C9—H9A	119.7
C13—N1—H1A	115.3	C11—C10—C9	119.2 (3)
C14—N1—H1A	115.3	C11—C10—H10A	120.4
C14—N2—C15	131.7 (2)	C9—C10—H10A	120.4
C14—N2—H2A	114.1	C10—C11—C12	120.4 (3)
C15—N2—H2A	114.1	C10—C11—H11A	119.8
O2—N3—O3	123.9 (3)	C12—C11—H11A	119.8
O2—N3—C18	117.7 (3)	C7—C12—C11	121.8 (3)
O3—N3—C18	118.5 (2)	C7—C12—H12A	119.1
C2—C1—C6	121.5 (3)	C11—C12—H12A	119.1
C2—C1—H1B	119.3	O1—C13—N1	121.5 (2)
C6—C1—H1B	119.3	O1—C13—C6	121.3 (2)
C1—C2—C3	121.7 (2)	N1—C13—C6	117.2 (2)
C1—C2—H2B	119.2	N2—C14—N1	113.8 (2)
C3—C2—H2B	119.2	N2—C14—S1	129.5 (2)
C4—C3—C2	116.2 (2)	N1—C14—S1	116.71 (19)
C4—C3—C7	122.0 (2)	C16—C15—N2	125.3 (2)
C2—C3—C7	121.8 (2)	C16—C15—C20	117.4 (2)
C5—C4—C3	122.5 (3)	N2—C15—C20	117.3 (2)
C5—C4—H4A	118.8	C17—C16—C15	120.7 (3)
C3—C4—H4A	118.8	C17—C16—H16A	119.6
C4—C5—C6	120.7 (2)	C15—C16—H16A	119.6
C4—C5—H5A	119.7	C18—C17—C16	119.9 (3)
C6—C5—H5A	119.7	C18—C17—H17A	120.1
C5—C6—C1	117.4 (2)	C16—C17—H17A	120.1
C5—C6—C13	125.5 (2)	C17—C18—C19	121.5 (3)
C1—C6—C13	117.1 (2)	C17—C18—N3	120.3 (3)
C8—C7—C12	116.7 (2)	C19—C18—N3	118.2 (3)
C8—C7—C3	121.9 (2)	C18—C19—C20	118.6 (3)
C12—C7—C3	121.4 (2)	C18—C19—H19A	120.7
C7—C8—C9	121.4 (3)	C20—C19—H19A	120.7
C7—C8—H8A	119.3	C19—C20—C15	121.8 (2)
C9—C8—H8A	119.3	C19—C20—Cl1	117.3 (2)
C10—C9—C8	120.5 (3)	C15—C20—Cl1	120.91 (19)
C10—C9—H9A	119.7		
			
C6—C1—C2—C3	−0.2 (5)	C5—C6—C13—N1	−6.0 (4)
C1—C2—C3—C4	1.0 (4)	C1—C6—C13—N1	175.1 (3)
C1—C2—C3—C7	−179.3 (3)	C15—N2—C14—N1	178.0 (2)
C2—C3—C4—C5	−0.7 (4)	C15—N2—C14—S1	−1.8 (4)
C7—C3—C4—C5	179.6 (3)	C13—N1—C14—N2	4.2 (4)
C3—C4—C5—C6	−0.5 (5)	C13—N1—C14—S1	−175.9 (2)
C4—C5—C6—C1	1.3 (4)	C14—N2—C15—C16	0.8 (5)
C4—C5—C6—C13	−177.5 (3)	C14—N2—C15—C20	−178.8 (3)
C2—C1—C6—C5	−0.9 (5)	N2—C15—C16—C17	−179.0 (3)
C2—C1—C6—C13	178.0 (3)	C20—C15—C16—C17	0.6 (4)
C4—C3—C7—C8	−179.0 (3)	C15—C16—C17—C18	1.0 (5)
C2—C3—C7—C8	1.3 (4)	C16—C17—C18—C19	−1.6 (5)
C4—C3—C7—C12	0.9 (4)	C16—C17—C18—N3	179.6 (3)
C2—C3—C7—C12	−178.8 (3)	O2—N3—C18—C17	1.9 (4)
C12—C7—C8—C9	0.5 (5)	O3—N3—C18—C17	−177.8 (3)
C3—C7—C8—C9	−179.7 (3)	O2—N3—C18—C19	−176.9 (3)
C7—C8—C9—C10	−0.8 (6)	O3—N3—C18—C19	3.3 (4)
C8—C9—C10—C11	0.6 (5)	C17—C18—C19—C20	0.5 (4)
C9—C10—C11—C12	−0.1 (5)	N3—C18—C19—C20	179.3 (2)
C8—C7—C12—C11	0.0 (5)	C18—C19—C20—C15	1.2 (4)
C3—C7—C12—C11	−179.8 (3)	C18—C19—C20—Cl1	−177.9 (2)
C10—C11—C12—C7	−0.2 (5)	C16—C15—C20—C19	−1.7 (4)
C14—N1—C13—O1	−4.7 (5)	N2—C15—C20—C19	177.9 (2)
C14—N1—C13—C6	173.9 (2)	C16—C15—C20—Cl1	177.3 (2)
C5—C6—C13—O1	172.6 (3)	N2—C15—C20—Cl1	−3.1 (3)
C1—C6—C13—O1	−6.2 (4)		

### Hydrogen-bond geometry (Å, º)

**Table:**

*D*—H···*A*	*D*—H	H···*A*	*D*···*A*	*D*—H···*A*
N2—H2*A*···Cl1	0.86	2.43	2.938 (2)	118
N2—H2*A*···O1	0.86	1.87	2.608 (3)	143
C16—H16*A*···S1	0.93	2.53	3.208 (3)	130
C10—H10*A*···O3^i^	0.93	2.57	3.354 (4)	142
C17—H17*A*···S1^ii^	0.93	2.77	3.673 (3)	165

Symmetry codes: (i) *x*+1, −*y*+3/2, *z*−1/2; (ii) −*x*+1, −*y*, −*z*+2.
